# Refining a Multicomponent Family-Based Health Education Package to Reduce Screen Time Among School-Aged Children: Qualitative Formative Evaluation With Parents and Caregivers

**DOI:** 10.2196/106521

**Published:** 2026-10-27

**Authors:** Mo Zhou, Yujie Yan, Siwei Sun, Hui Yin

**Affiliations:** 1 The Second People's Hospital of Shenzhen and First Affiliated Hospital of Shenzhen University, Shenzhen, China; 2 China Japan Friendship Hospital, Beijing, China; 3 Peking University China-Japan Friendship School of Clinical Medicine, Beijing, China; 4 Peking University Sixth Hospital, Peking University Institute of Mental Health, NHC Key Laboratory of Mental Health (Peking University), National Clinical Research Center for Mental Disorders (Peking University Sixth Hospital), Beijing, China; 5 School of Public Health, Peking University, No. 38 Xueyuan Road, Haidian District, Beijing, 100191, China, 86 1082805853; 6 Institute for Global Health, Peking University, Beijing, China

**Keywords:** screen time, school-aged children, caregivers, family-based intervention, health education, formative evaluation, qualitative research, digital media

## Abstract

**Background:**

Excessive screen time among school-aged children is a growing concern for child health and family routines. A previous systematic review of children aged 6 to 14 years found that excessive screen time is common in this age group, highlighting the need for feasible intervention strategies that can be implemented in daily family routines. Although many health education materials communicate the harms of excessive screen use, families often need practical support to translate risk messages into household rules, parent-child communication, alternative activities, and changes to the home environment.

**Objective:**

This study aimed to conduct a qualitative formative evaluation of a preliminary multicomponent family-based health education package designed to support healthier screen-use practices among school-aged children, with a primary focus on reducing excessive recreational screen time and associated family routines, and to identify refinements needed before pilot implementation.

**Methods:**

Semistructured online video interviews were conducted with 10 parents or primary caregivers of school-aged children aged 6 to 12 years from October to December 2024. The package included caregiver education for parents and grandparents, child-facing materials such as a picture book and posters, home-use reminder cues, and home-environment guidance through an online self-assessment tool that generated tailored household action suggestions. The tasks assigned to the tool were limited to these functions. Participants reviewed the package components and commented on clarity, acceptability, perceived usefulness, feasibility, fit with family routines, and suggestions for refinement. Data were analyzed using directed content analysis and thematic analysis, informed by the Theoretical Framework of Acceptability (TFA) and health communication material-evaluation dimensions.

**Results:**

Four themes were generated. First, the package was generally regarded as acceptable to the participating caregivers, but comprehension did not always translate into self-reported immediate recall during interviews. Second, concrete, scenario-based messages were described as more meaningful than abstract risk information. Third, caregivers wanted action guidance rather than additional risk information. Fourth, caregivers preferred practical, age-adapted, and home-embedded tools that could be repeatedly encountered in daily routines.

**Conclusions:**

Among this selected group of caregivers, the preliminary multicomponent family-based screen-time education package was generally perceived as understandable and acceptable, but participant-informed refinements are needed before pilot implementation. The findings suggest that the package should move beyond general risk communication toward a family action toolkit that combines caregiver guidance, child-facing materials, concrete consequence chains, parent-child negotiation supports, replacement activity lists, and home environmental cues.

## Introduction

### Background

Excessive screen time among school-aged children has become a growing public health concern. A systematic review of children aged 6 to 14 years found a pooled mean screen time of 2.77 hours per day (0.77 h above the 2-h reference value; 95% CI 0.32-1.22) and that 46.4% of school-aged children had an average screen time of at least 2 hours per day [1]. These findings suggest that excessive screen time is common in this age group and highlight the need for feasible intervention strategies that can be implemented in daily family routines. Current public health guidance also recommends limiting sedentary time, particularly recreational screen time, while maintaining sufficient physical activity and sleep [2,3]. The Canadian 24-Hour Movement Guidelines for Children and Youth, for example, recommend no more than 2 hours per day of recreational screen time for children and youth aged 5 to 17 years [4].

For school-aged children, screen-time management is not simply an individual behavior-change issue. It is embedded in family routines, school-related learning, entertainment, peer interaction, and household supervision. Parents and other caregivers often act as gatekeepers of children’s screen use through rule-setting, device access, supervision, modeling, and organization of alternative activities. Family media-planning approaches similarly emphasize household rules, screen-free spaces, bedtime boundaries, and family-specific guidance rather than generic warnings alone [5,6].

Prior qualitative work suggests that parents manage their children’s screen viewing by linking rules to daily events such as meals, before and after school, and bedtime, while also struggling with contemporary screen environments and the need to achieve digital balance in family life [7-10]. These challenges suggest that family-based screen-time interventions should move beyond general risk communication. A multicomponent approach may be needed to combine caregiver education, child-facing materials, home environmental cues, and household implementation tools.

Formative evaluation is particularly important before pilot implementation of such interventions. Consistent with updated Medical Research Council guidance, early development work for complex interventions should clarify how an intervention is understood, used, and refined before definitive evaluation [11]. Acceptability, comprehensibility, perceived usefulness, and feasibility may shape whether families can use intervention materials as intended. The Theoretical Framework of Acceptability (TFA) conceptualizes acceptability through multiple constructs, including affective attitude, burden, perceived effectiveness, ethicality, intervention coherence, opportunity costs, and self-efficacy [12]. Applying such a framework during early-stage development can help identify not only whether caregivers understand or like a package but also whether the package fits family routines and supports action.

### Objective

A preliminary multicomponent family-based health education package was developed to support healthier screen-use practices among school-aged children, with a primary focus on reducing excessive recreational screen time and related family routines. The package combined caregiver education, child-facing materials, home reminder cues, and home environment guidance. Several components also addressed the quality, context, and safety of screen use, including family interaction during screen use, mealtime routines, privacy protection, online strangers, and cyberbullying. These topics were included not as separate intervention end points but as developmentally appropriate supports for healthier family screen-use practices, especially for older children whose screen use increasingly involves social, informational, and online-safety dimensions.

This study aimed to conduct a qualitative formative evaluation of the package with parents and primary caregivers. Specifically, we examined how caregivers understood and perceived the package components, what messages were more or less consistently recalled during interviews, what implementation gaps were identified, and how the package should be refined before pilot testing.

## Methods

### Study Design

We conducted a qualitative formative evaluation of a preliminary multicomponent family-based health education package designed to support healthier screen-use practices among school-aged children in home settings, with a primary focus on reducing excessive recreational screen time and related family routines. The purpose was not to estimate intervention effectiveness but to assess caregivers’ understanding, acceptability, perceived usefulness, feasibility, and suggestions for package refinement before pilot implementation. Reporting was guided by the COREQ (Consolidated Criteria for Reporting Qualitative Research), and the package components were described using principles consistent with the Template for Intervention Description and Replication (TIDieR) checklist [13,14]. The completed COREQ checklist is provided in Checklist 1.

### Health Education Package

The preliminary intervention was designed as a multicomponent family-based health education package. It was not a stand-alone video intervention. The package combined 4 components.

First, the caregiver lecture component included parent and grandparent versions, delivered through offline lectures with online video replay. It included 8 core topics: harms of excessive screen time, setting screen-time rules, screen time and eye health, screen time and sleep health, screen time and physical activity, mealtime screen use, home placement of digital devices, and reinforcement sessions. The 8 core topics were selected through an iterative development process that combined evidence on children’s screen time and sedentary behavior, public health and pediatric guidance on screen-time management, family media-planning principles, and the research team’s prior work on screen-time patterns among school-aged children. The topics were organized to link health risks with modifiable family practices. For example, eye health, sleep health, and physical activity addressed commonly recognized health consequences of excessive screen use; screen-time rules, mealtime screen use, and device placement addressed household routines and environmental control; reinforcement sessions were included to support repeated exposure and caregiver implementation.

Second, the child-facing educational component included a picture book and posters. The child-facing materials were age-adapted for children aged 6 to 8 years, 9 to 12 years, and 13 to 14 years, and covered messages such as limiting screen time, taking breaks, avoiding screens before bedtime or while walking, increasing outdoor activities, avoiding screens during meals, following family rules, protecting privacy, avoiding strangers online, and seeking help for cyberbullying. The child-facing materials were age-adapted to reflect developmental progression. Materials for children aged 6 to 8 years emphasized simple rules, visual cues, and concrete daily situations. Materials for children aged 9 to 12 years included more explanation of consequences, family rules, and self-management. Materials for children aged 13 to 14 years introduced greater autonomy and digital-safety topics, including privacy, online strangers, and cyberbullying. Because the interviewed caregivers had children aged 6 to 12 years, feedback on materials intended for 13- to 14-year-olds was interpreted only as indirect caregiver feedback and as a refinement issue for future pilot work.

Third, the home-use reminder component included daily household cues such as umbrellas reminding children not to look at mobile phones when crossing the road, table mats reminding families not to use screens during meals, door hangers reminding children not to use mobile phones 1 hour before bedtime, and sticky notes encouraging children to look up and observe the sky.

Fourth, the home-environment guidance component consisted of an individualized online self-assessment tool. The tool assessed 6 domains of the family screen environment: physical environment, time rules, content supervision, joint screen use, caregiver modeling, and safety awareness. It generated graded feedback and rule-based tailored suggestions, such as bedroom device rules, screen-free areas, device-return routines, caregiver modeling, replacement activities, and online-safety conversations. The tool was intended to support caregivers in translating general guidance into concrete household actions.

A component-level description of the preliminary health education package is provided in Multimedia Appendix 1.

### Participants and Recruitment

Participants were recruited through a study announcement posted on the Wenjuanxing (wjx.cn; Changsha Ranxing Information Technology Co, Ltd) online survey platform. Eligible participants were adults aged 18 years or older who were primary caregivers of a school-aged child, were physically healthy, and reported no psychiatric or neurological conditions affecting their capacity to participate in the interview.

By the enrollment cutoff, 167 caregivers had expressed interest in being interviewed. Before interviews began, the research team created a sampling matrix using available screening information from interested caregivers. The matrix considered region, caregiver role, caregiver age, number of children, household size, family structure, and whether grandparents or other relatives were involved in caregiving. Two members of the research team reviewed the screening information and purposively invited participants to achieve maximum variation in caregiving contexts. The purpose of maximum-variation sampling was to broaden the range of family situations represented in this focused formative evaluation, not to enable subgroup comparisons by participant characteristics.

The target sample size of approximately 10 interviews was determined in advance because the study had a narrow, package-specific aim, the interviews were expected to generate detailed component-level feedback, and the analysis was intended to identify refinement needs rather than compare groups, consistent with the information power approach to qualitative interview sample size [15]. After 10 interviews, the research team judged that sufficient information power had been reached because feedback repeatedly converged around actionability, concrete examples, age adaptation, and home-embedded supports.

The final sample included 10 caregivers: 8 mothers, 1 father, and 1 uncle serving as a primary caregiver. Their children were aged 6 to 12 years old and were enrolled in grades 1 to 5 of primary school. Family contexts included nuclear families, multigenerational households, and kinship or left-behind caregiving arrangements. Each participant received RMB 200 (RMB 1=US $0.14 as of December 2, 2024) for their time. Because the achieved sample included mostly mothers, variation in caregiver sex was limited. The findings should therefore not be interpreted as supporting comparisons by caregiver sex or as broadly transferable across all caregiver groups.

### Data Collection

Semistructured interviews were conducted between October and December 2024 via online video calls on Tencent Meeting. All interviews were conducted by a trained graduate student interviewer. The interviewer had participated in baseline survey design and implementation but was not involved in developing the health education materials. Participants had no prior relationship with the interviewer. Each interview lasted approximately 60 minutes, and the package-evaluation section occupied most of the interview time.

The interview guide included 3 sections: experience completing the baseline questionnaire, experience with family screen-time supervision, and evaluation of the health education package. This manuscript reports only the package-evaluation section; the other sections were used to understand each family’s caregiving context. The package-evaluation section usually lasted approximately 45 to 60 minutes and covered information uptake, comprehensibility, conciseness, perceived guidance, attractiveness, overall impression, appropriateness, and suggestions for improvement. The English version of this section is provided in Multimedia Appendix 2.

The package components were presented in the same order for all participants: caregiver education, child-facing materials, home-use reminder cues, and home-environment guidance. Participants reviewed selected examples rather than the full package. For the caregiver education component, short video clips from the parent and grandparent lectures were shown. For the child-facing component, electronic posters were shown via screen sharing, and the printed picture book was shown on camera. For the home-use reminder component, photographs of the table mat, door hanger, umbrella, and sticky notes were shown. The online home-environment self-assessment tool was described verbally rather than used interactively during the interview. Feedback on this component was therefore interpreted as feedback on the tool’s concept, intended function, and perceived usefulness rather than feedback based on hands-on use.

Self-reported immediate recall was assessed informally during the package-evaluation discussion. After participants viewed or heard about each component, the interviewer asked which messages they remembered most clearly, which parts left a weaker impression, and what they thought should be revised. This procedure was used to explore perceived salience and recall during the interview. It was not designed or interpreted as a formal memory assessment.

With participants’ permission, interviews were audio-recorded. The interviewer also took field notes during and immediately after each interview. The recordings were transcribed using the transcription function of Tencent Meeting, and the transcripts were manually checked and corrected against the recordings by the research team.

### Data Analysis

Transcripts from the package-evaluation section were analyzed using directed content analysis and reflexive thematic analysis. Directed content analysis provided the initial coding structure, while thematic analysis was used to identify patterns not fully captured by the initial framework [12,16,17].

The initial framework was developed from the TFA and the health communication material-evaluation dimensions. TFA constructs were used to code acceptability-related responses, including affective attitude, burden, perceived effectiveness, ethicality, intervention coherence, opportunity costs, and self-efficacy. Health communication dimensions were used to code comments on message comprehension, self-reported immediate recall during interviews, actionability, attractiveness, and appropriateness. These deductive codes helped organize responses to the package components and identify whether participants understood, accepted, and considered the materials useful in family routines.

Inductive coding was then used to capture issues that were raised by participants but were not sufficiently specified in the initial framework. These included hyperfocus as a recognizable family scenario, difficulty distinguishing learning-related and recreational screen use, conflict around returning devices, the need for replacement activities, and preferences for routine-embedded objects such as table mats, stationery, and notebooks. Candidate inductive codes were compared across participants and then grouped with deductive codes to develop higher-level themes.

The final 4 themes were developed by grouping codes according to the main refinement problem they identified. Theme 1 was primarily informed by deductive codes on acceptability, comprehension, and self-reported immediate recall. Themes 2 and 3 combined deductive material-evaluation codes with inductive codes on concrete scenarios, consequence chains, and action guidance. Theme 4 was mainly developed from inductive codes on age adaptation, daily-use objects, and fit with household routines, while also drawing on deductive codes related to appropriateness, burden, and perceived usefulness. Participant counts reported for each theme indicate the spread of supporting evidence across the sample. They are not intended as prevalence estimates or as evidence of generalizability.

Coding proceeded in 3 stages. First, 2 researchers independently read the transcripts and applied the initial coding framework. Second, ChatGPT, using GPT-5.5 Thinking through the OpenAI web interface, was used in June and July 2026 as a supplementary organizational aid. The tasks assigned to the tool were limited to organizing deidentified qualitative excerpts by provisional topic and supporting English-language editing. Selected deidentified meaning units from the package-evaluation section were submitted. These consisted of short excerpt-level units rather than full transcripts and were used only for provisional grouping and language organization. Full transcripts, audio recordings, participant names, contact information, locations, institutions, and the raw reconciliation spreadsheet were not submitted. AI-generated suggestions were treated as provisional organizational outputs, not as evidence or independent analytic confirmation. Third, the 2 researchers compared their coding, discussed discrepancies, refined the codebook, finalized themes, and selected quotations directly from the verified transcripts. Suggestions generated by AI were corrected, discarded, or incorporated only when they were supported by the original transcript data.

Coded excerpts, code definitions, analytic memos, theme matrices, and package-refinement recommendations were managed in Microsoft Word and Excel. Candidate quotations were first selected in Chinese from the verified transcripts and anonymized by removing names, locations, institutions, and other potentially identifying details. They were then translated into English by a bilingual member of the research team and checked against the original Chinese transcripts by a second bilingual researcher. Quotations were lightly edited for readability only when necessary, for example, by removing fillers, repeated words, or incomplete sentence fragments, without changing the participant’s meaning or voice. Omissions are indicated with ellipses, and clarifying words are inserted in brackets where needed to preserve readability or protect anonymity. The original Chinese quotations are not published to reduce the risk of identifiability, but they remain stored in the secure research records for verification. The coding framework, theme development process, reconciliation information, and participant-level evidence coverage are provided in Multimedia Appendix 3.

### Reflexivity and Trustworthiness

Several strategies were used to strengthen the credibility, dependability, confirmability, and transferability of the analysis. Credibility was supported by using a semistructured interview guide aligned with the package components, checking candidate quotations against the original transcripts, and selecting quotations from multiple participants to reflect different types of feedback. Dependability was supported by documenting code definitions, coding decisions, analytic memos, theme development, and reconciliation decisions in Microsoft Word and Excel. Confirmability was strengthened by independent coding before reviewing AI-generated suggestions, comparison and discussion of coding discrepancies, and explicit attention to the analytic team’s prior involvement in package development. The interviewer was not a member of the material-development team and had no prior relationship with participants. Transferability was supported by describing participant characteristics, caregiving contexts, materials-exposure procedures, and the limits of the selected sample. Additional details on coding comparison, reconciliation decisions, and participant-level evidence coverage are reported in Multimedia Appendix 3.

Coding comparison was used as a diagnostic process to identify and resolve differences in interpretation rather than as a primary criterion for validity. Disagreements mainly concerned whether a meaning unit should be treated as theme-relevant or as background context, and how detailed the coding should be. These disagreements did not lead to conflicting substantive interpretations of the final themes. Disagreements were resolved through discussion, codebook clarification, and checking against the original transcripts. The analysis avoided treating positive comments, stated willingness, or perceived usefulness as evidence of intervention effectiveness.

### Ethical Considerations

This study was reviewed and approved by the Peking University Health Science Center Ethics Committee (IRB00001052-21158). Before each interview, participants received information about the study purpose, interview procedures, voluntary participation, confidentiality, and their right to withdraw at any time. Because the interviews were conducted through online video calls, oral informed consent was obtained and audio-recorded before data collection; this oral consent procedure was approved by the ethics committee. Participants consented to the publication of anonymized quotations, and the manuscript contains no identifiable personal information. Each participant received RMB 200 as compensation for their time.

Interview recordings and transcripts were stored securely and were accessible only to the research team. Transcripts were deidentified before analysis and reporting, and quotations were anonymized by removing names, locations, institutions, and other potentially identifying details. The ethics approval and consent procedures permitted the use of anonymized qualitative excerpts for research analysis and reporting, including supplementary AI-assisted organization of deidentified excerpts. For AI-assisted processing, only deidentified excerpts were used; full transcripts, audio recordings, participant identifiers, and the raw reconciliation spreadsheet were not submitted to the AI tool. Human researchers made all final decisions on coding, theme development, quotation selection, interpretation, and manuscript content.

## Results

### Participant Characteristics

Ten parents or primary caregivers participated. The children were aged 6 to 12 years and enrolled in grades 1 to 5 of primary school. Participants included mothers, one father, and one uncle who served as a primary caregiver. Family contexts varied, including nuclear families, multigenerational households with grandparent involvement, and kinship-based or left-behind caregiving arrangements. Table 1 summarizes participant characteristics using broad categories to reduce identifiability.

**Table 1.** Participant characteristics.

Participant^a^	Primary caregiver	Child school stage	Family context	Screen-use and supervision context
P01	Mother	Upper primary	Nuclear family	Weekday recreational screen use restricted; weekend television and tablet use; timer and verbal supervision
P02	Mother	Lower primary	Multigenerational involvement	Learning app use in short sessions; weekend use somewhat longer; prior agreements and forced shutdown used when needed
P03	Mother	Middle primary	Multigenerational household	Limited television use; mobile phone use restricted; phone-based time limits and prior agreements
P04	Mother	Upper primary	Nuclear family	Tablet use approximately 1 hour; verbal supervision and system time controls
P05	Mother	Lower primary	Multigenerational involvement	Minimal weekday use; weekend use allowed with coviewing and prior agreement
P06	Mother	Middle primary	Multigenerational household	Short-video or tablet use approximately 1 hour; safety software and reminders
P07	Father	Lower primary	Multigenerational household	Brief phone-based music use; timer and caregiver control of devices
P08	Uncle	Upper primary	Kinship caregiving context	Gaming with peer interaction; partial supervision and grandparent involvement
P09	Mother	Lower primary	Multigenerational household	Limited backup phone use on weekdays; longer weekend use when unsupervised; device taken back before bedtime
P10	Mother	Upper primary	Nuclear family	Learning tablet use and increased holiday use; duration control with content-monitoring challenges

a P01-P10 represent individual participants (parents or primary caregivers).

### Themes

#### Overview

Four themes were generated. First, the package was generally regarded as acceptable by the participating caregivers, but comprehension did not always translate into self-reported immediate recall during interviews. Second, concrete, scenario-based messages were described as more meaningful than abstract risk information. Third, caregivers wanted action guidance rather than additional risk information. Fourth, caregivers preferred practical, age-adapted, and home-embedded tools. Figure 1 summarizes the formative evaluation logic and refinement pathway, and Table 2 presents representative quotations. A participant-level summary of coded evidence across the 4 themes is provided in Multimedia Appendix 3. Additional supporting quotations are provided in Multimedia Appendix 4.

**Figure 1.**
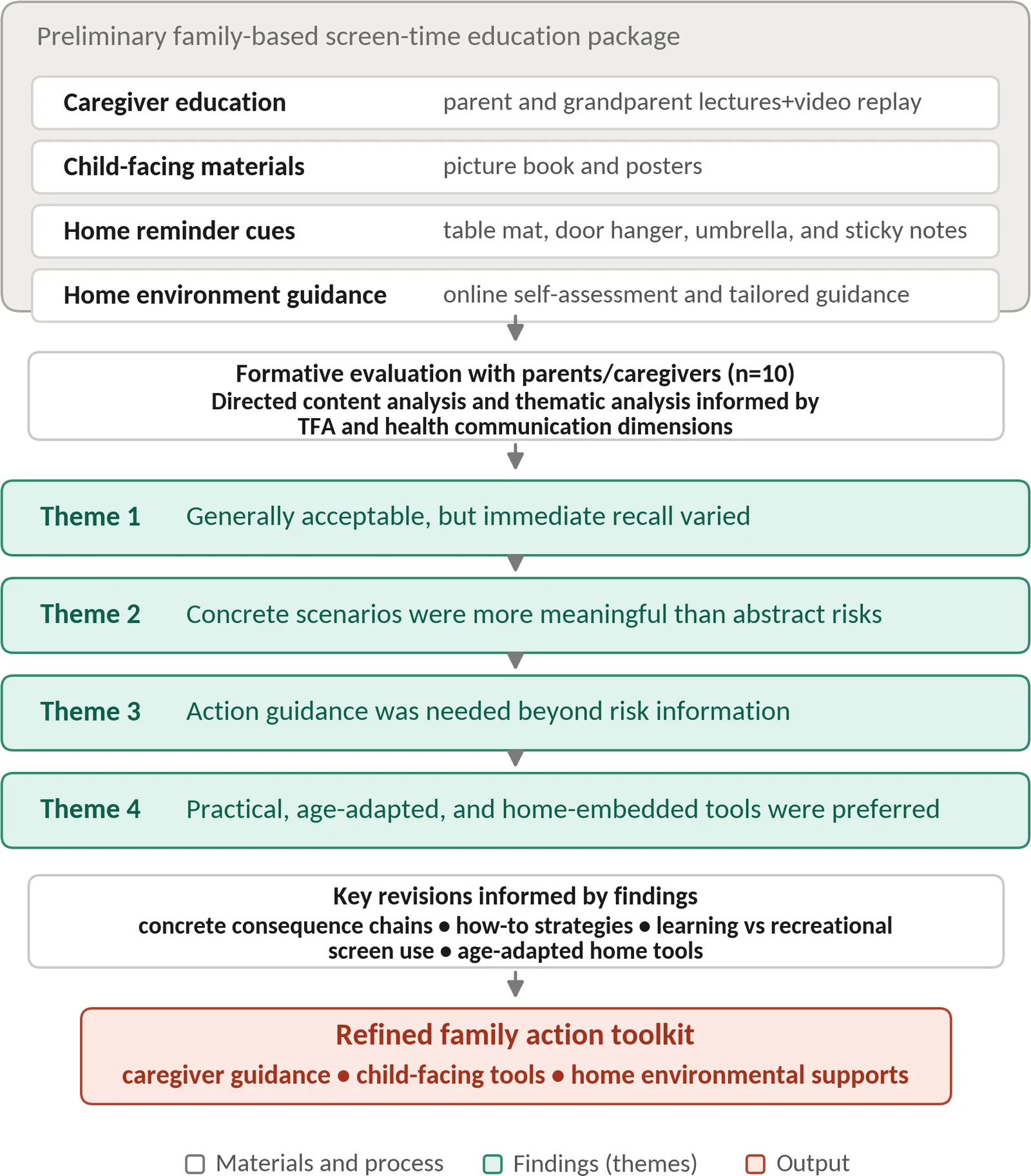
Formative evaluation logic and refinement pathway for the multicomponent family-based screen-time education package. TFA: Theoretical Framework of Acceptability.

**Table 2.** Themes and representative quotations.

Theme	Analytic meaning	Representative quotation^a^
Theme 1: the package was generally acceptable, but comprehension did not always translate into immediate recall	Participants generally found the materials understandable, but some had difficulty recalling specific information after viewing.	“It was easy to understand...but when you asked me again, the impression was not that deep...I watched it, but when you asked me, I could not really say it.” (P03) “The harms were mentioned earlier, but the impression was not deep.” (P05) “The video was easy to understand, plain, and clear at a glance.” (P02)
Theme 2: concrete, scenario-based messages were described as more meaningful than abstract risk information	Messages connected to familiar family, school, road-safety, or gaming scenarios appeared to be more meaningful than general risk statements.	“There was this idea of hyperfocus. My [child] is exactly like that. Whenever [the child] plays with electronic devices, no matter how much the whole family talks, [the child] cannot hear us.” (P01) “The impact was not strong…I need something more impactful, whether in the images or the content.” (P06) “Some real reports could be added...for example, holding a phone while crossing the road and being hit by a car. These real cases do happen.” (P05) “The harms section could be longer and include examples...some children play games and spend family money.” (P09)
Theme 3: caregivers wanted action guidance rather than additional risk information	Participants wanted concrete methods for rule-setting, time control, negotiation, managing resistance, and replacement activities.	“Parents mostly understand these harms...but some do not know what to do. The specific measures on how to do it could be expanded.” (P08) “What parents need more is ‘how to do it.’...the part on hyperfocus was a bit long, but the part on how to help children avoid that state was very short.” (P05) “It said screen time should not exceed 2 hours and 30 minutes each time, but how do we control it? It could list several methods.” (P10)
Theme 4: caregivers preferred practical, age-adapted, and home-embedded tools	Participants preferred reminder materials that could be repeatedly encountered in daily life and adjusted to children’s age and literacy level.	“This kind of form is not very meaningful. Many people may just take it and throw it away...It would be better to make a small notebook or pencil case for [the child], with some illustrations.” (P07) “For children, things like rulers, pens, and stationery, small items that can be carried around and used every day, would be better.” (P04) “The table mat is good...because everyone eats every day, so they can see it every day.” (P06)

a P01-P10 represent individual participants (parents or primary caregivers).

#### Theme 1: The Package Was Generally Acceptable, but Comprehension Did Not Always Translate Into Immediate Recall

Coded excerpts supporting this theme were identified in 9 of 10 participants. Most participants generally considered the package understandable and acceptable. For example, P02 described the material as “easy to understand, plain, and clear at a glance.” However, understanding the material during exposure did not necessarily mean that specific messages were later recalled during the interview. P03 described this gap directly: “It was easy to understand...but when you asked me again, the impression was not that deep...I watched it, but when you asked me, I could not really say it.” P05 similarly noted that the harms had been mentioned but that “the impression was not deep.”

This theme refers to self-reported immediate recall during interviews rather than on a formal memory test. The pattern suggested that some messages were clear at the moment of viewing but were not sufficiently memorable or prioritized for later recall.

#### Theme 2: Concrete, Scenario-Based Messages Were Described as More Meaningful Than Abstract Risk Information

Coded excerpts supporting this theme were identified among 7 of 10 participants. The term “hyperfocus” was used in the materials to describe a state in which a child becomes highly absorbed in screen use and becomes difficult to interrupt or redirect. This idea appeared to map onto familiar family experiences. P01 said, “There was this idea of hyperfocus. My [child] is exactly like that. Whenever [the child] plays with electronic devices, no matter how much the whole family talks, [the child] cannot hear us.” P03 similarly described a holiday experience in which the child watched screens “very intensely” and the caregiver did not know how to manage it.

Participants also wanted general harms to be connected to visible consequences. P01 suggested that vision-related harm could be made more concrete by explaining that poor eyesight may make it difficult to see the blackboard and then affect learning. P05 suggested adding real reports, such as reports of children holding phones while crossing the road and being hit by a car. P09 suggested adding examples related to gaming and spending family money. These comments did not indicate that participants rejected abstract risk information; rather, they wanted it to be translated into concrete, everyday consequence chains.

#### Theme 3: Caregivers Wanted Action Guidance Rather Than Additional Risk Information

Coded excerpts supporting this theme were identified in 7 of 10 participants. Many caregivers felt that families already understood the harms of excessive screen use but lacked concrete methods for family implementation. P08 stated, “Parents mostly understand these harms...but some do not know what to do. The specific measures on how to do it could be expanded.” P05 made a similar point: “What parents need more is ‘how to do it’...the part on hyperfocus was a bit long, but the part on how to help children avoid that state was very short.”

Participants described specific implementation questions: how to control the duration, how to help children return devices at the agreed time, how to manage resistance, how to distinguish learning-related and recreational screen use, and how to provide alternative activities that are low cost and attractive to children. P10 illustrated this need for operational detail: “It said screen time should not exceed 2 hours and 30 minutes each time, but how do we control it? It could list several methods.”

#### Theme 4: Caregivers Preferred Practical, Age-Adapted, and Home-Embedded Tools

Coded excerpts supporting this theme were identified in 8 of 10 participants. Participants did not reject reminder materials as a category; instead, they differentiated between generic publicity items and tools that could be embedded in daily family routines. P06 viewed the table mat positively because “everyone eats every day, so they can see it every day.” P08 also noted that reminder materials could prompt caregivers when they forgot to manage their child’s screen use.

At the same time, several participants suggested that some materials should be redesigned into more practical, age-appropriate objects. P07 said that some publicity items might be taken and thrown away and suggested making “a small notebook or pencil case for [the child], with some illustrations.” P04 similarly suggested “rulers, pens, and stationery” because they are small, portable, and used every day. Participants also raised age-adaptation issues, including whether picture books were more suitable for younger children; whether pinyin, the phonetic spelling system for Chinese characters, or more visuals were needed; and whether older children required materials that respected their autonomy and addressed online safety.

### Formative Refinement Implications

Table 3 translates the 4 themes into formative component-level refinement implications. These implications are derived from caregiver feedback and are interpreted as formative recommendations for package development rather than as evidence of effectiveness.

**Table 3.** Formative component-level refinements derived from caregiver feedback.

Package component	Intended function	Caregiver feedback from formative evaluation	Proposed refinement
Caregiver education	Improve caregiver understanding and skills for managing screen time at home.	Caregivers understood risk messages but wanted more “how-to” strategies for rules, negotiation, duration control, and resistance management.	Add scenario-based examples, rule-setting templates, negotiation scripts, device-return strategies, and troubleshooting guidance.
Child-facing materials	Provide age-adapted messages that children can understand and discuss with caregivers.	Caregivers valued child-facing materials but noted concerns about readability, visual design, and age fit.	Develop age-graded versions; reduce text density; add visuals, pinyin where appropriate, parent-child discussion prompts, and older-child content on autonomy, privacy, online safety, and cyberbullying.
Home reminder cues	Prompt action at the point of daily decision-making.	High-frequency items such as table mats were valued; generic publicity items were viewed as easy to ignore or discard.	Prioritize practical daily-use items such as table mats, bedtime cards, desk prompts, notebooks, pencil cases, rulers, and stationery.
Home environment guidance	Support modification of the household screen environment.	Caregivers valued concrete standards but wanted clearer guidance on shared spaces, device placement, and bedroom rules.	Add room-by-room checklists, device-placement prompts, screen-free-zone guidance, bedroom device rules, and tailored feedback from the self-assessment tool.
Overall package	Integrate components into a coherent family implementation support package.	The main gap was translating risk awareness into daily action.	Refine the package into a family action toolkit combining caregiver guidance, child-facing materials, home cues, environmental supports, replacement activity lists, and family media planning tools.

## Discussion

### Principal Findings

This qualitative formative evaluation found that the preliminary multicomponent family-based screen-time education package was generally regarded as acceptable by participating caregivers, but refinement is needed before pilot implementation. The central finding was that caregivers did not simply need more information about the harms of excessive screen use. Rather, they needed practical support to translate risk messages into family rules, parent-child communication, replacement activities, and environmental cues embedded in daily routines. The findings indicate that the package should be refined from a risk-communication package into a more actionable family toolkit.

### Comparison With Prior Work

This study builds on prior evidence that excessive screen time is common among school-aged children [1]. The present study extends prior work beyond describing the burden and patterns of screen time to refining a family-based health education package intended for later pilot implementation.

Our findings align with qualitative and review evidence showing that parents are central to children’s screen-time management but often face practical barriers when translating general recommendations into daily routines. A qualitative meta-synthesis found that parents have concerns and confusion about children’s screen time and use varied strategies to manage it [7]. Solomon-Moore et al [8] highlighted the challenges posed by contemporary screen environments and the need to achieve digital balance in family life. Our study extends this literature by examining how such concerns arise when caregivers review a concrete multicomponent package.

The importance of embedding screen-time guidance in everyday routines is also supported by prior qualitative work. Jago et al [9] found that parents managed screen viewing by setting limits around daily events such as meals, before and after school, and bedtime. This is consistent with our finding that home reminder cues were valued when they were linked to repeated household routines, such as dining, bedtime, walking outdoors, or study settings. Rather than functioning as generic publicity items, these cues may be more useful when they serve as prompts at the point where family decisions about screen use are made.

Our findings also support the idea that family screen-time interventions require action-oriented components, not only risk communication. A qualitative metasynthesis on reducing youth screen time concluded that mutually agreed rules, limits, and parental monitoring were perceived as likely facilitators, and parents themselves could model screen engagement [10]. Systematic review evidence on interventions to reduce children’s screen time also emphasizes the need to identify effective intervention strategies and behavior-change components [18]. From a behavior-change perspective, participants’ feedback pointed to gaps not only in knowledge but also in capability, opportunity, and motivation to implement screen-time rules in daily routines [19].

### Refinement Implications

The findings can be interpreted through the TFA. Intervention coherence appeared relatively strong because caregivers generally understood the package and found many messages clear. However, self-efficacy and perceived opportunity appeared more limited: caregivers knew that screen time should be managed but wanted more practical support to do so. Opportunity costs were also implicit in requests for low-cost replacement activities and realistic family routines.

Table 3 summarizes the main component-level refinements derived from caregiver feedback. In brief, the package should make risk messages more concrete, strengthen operational guidance for caregivers, and redesign reminders as practical daily-use tools with stronger age adaptation.

### Future Pilot Implementation

The refined package should be tested in a future pilot study before a definitive effectiveness evaluation. A pilot study should assess recruitment and retention feasibility, delivery feasibility across family and school-community settings, acceptability among caregivers and children, completion and use of package components, and fidelity to core messages. Preliminary implementation outcomes may include adoption of family screen-time rules, parent-child communication about screen use, use of replacement activities, uptake of home environmental modifications, and caregiver confidence in managing recreational screen time. Exploratory behavioral outcomes could include parent-reported recreational screen time, bedtime screen use, mealtime screen use, and device use in bedrooms. The formative findings from this study provide a basis for specifying the pilot intervention content, delivery channels, implementation support, and outcome measures.

### Strengths and Limitations

Strengths of this study include evaluation of a real multicomponent package before pilot implementation, use of in-depth interviews, inclusion of several caregiving contexts, and verification of quotations against original transcripts. The analysis was transparent about the role of AI-assisted preliminary organization and retained human responsibility for all analytic decisions. Limitations include the small selected sample, recruitment from an online parent community, limited variation in caregiver sex, reliance on caregiver perspectives, and absence of child, grandparent, teacher, or health professional interviews. Because several authors developed the package and participants reviewed the materials with a researcher present after receiving compensation, positive appraisals may partly reflect courtesy or social-desirability bias; findings on acceptability should be read with this in mind. The sample may have overrepresented caregivers who were already motivated to manage children’s screen use, as several participants reported using restrictions, timers, supervision, or device-control strategies before the interview. Because participants reviewed selected examples of a researcher-presented prototype rather than using the complete package independently at home, the findings reflect perceived acceptability and refinement needs under interview conditions. In addition, although some package materials included content for children aged 13 to 14 years, the interviewed caregivers had children aged 6 to 12 years; suggestions about older-child adaptation should therefore be treated as a refinement implication to be tested in future work rather than as a directly sampled perspective. The findings should be interpreted as formative insights rather than evidence of effectiveness. Online presentation of materials may also differ from how families would engage with the package in actual home, school, or community settings.

### Conclusions

Among this selected group of caregivers, the preliminary package was generally perceived as understandable and acceptable, but participants identified important refinements needed before pilot implementation. The key gap was not whether caregivers understood that excessive screen use could be harmful, but whether the package gave them enough practical support to put into practice daily family routines. Future versions should move toward a family action toolkit that combines caregiver guidance, child-facing tools, concrete consequence chains, parent-child negotiation support, replacement activity lists, and home environmental cues.

## Supplementary material

10.2196/106521 Multimedia Appendix 1 Description of the preliminary multicomponent family-based health education package.

10.2196/106521 Multimedia Appendix 2 Semistructured interview guide used to evaluate the health education materials.

10.2196/106521 Multimedia Appendix 3 Coding framework and theme development process.

10.2196/106521 Multimedia Appendix 4 Additional representative quotations supporting the 4 qualitative themes identified in the formative evaluation.

10.2196/106521 Checklist 1 COREQ checklist.

## Abbreviations

COREQ: Consolidated Criteria for Reporting Qualitative Research
TFA: Theoretical Framework of Acceptability
TIDieR: Template for Intervention Description and Replication
