# Supplementary material for: Refining a Multicomponent Family-Based Health Education Package to Reduce Screen Time Among School-Aged Children: Qualitative Formative Evaluation With Parents and Caregivers
Source: JMIR Form Res. 2026 Oct 27;10:e106521. doi: 10.2196/106521 (PMC13712257; doi:10.2196/106521)
Supplement: Supplementary file 1 [file formative-v10-e106521-s001.pdf]

# Multimedia Appendix 2

## Preliminary Health Education Package Components

*This appendix describes the preliminary multicomponent family-based health education package evaluated in the qualitative formative study. The package was designed to support healthier screen-use practices among school-aged children, with a primary focus on reducing excessive recreational screen time and related family routines. Several components also addressed the quality, context, and safety of screen use, including family interaction, mealtime routines, privacy protection, online strangers, and cyberbullying. The package was preliminary and pre-pilot. It was not a stand-alone video intervention and did not make claims about reducing screen time.*

## Overview

The package was designed to support healthier screen-use practices among school-aged children by combining caregiver education, child-facing materials, home reminder cues, and home-environment guidance. Its primary focus was reducing excessive recreational screen time and related family routines, while also addressing developmentally relevant issues related to the context, quality, and safety of screen use. The intended logic was to translate risk communication into practical family action by linking information, reminders, household routines, caregiver guidance, child-facing materials, and environmental supports.

## **Development** Rationale

The eight core caregiver-education topics and four package components were selected through an iterative development process that combined evidence on children's screen time and sedentary behavior, public health and pediatric guidance on screen-time management, family media-planning principles, and the research team's prior work on screen-time patterns among school-aged children. The topics were organized to link health risks with modifiable family practices. Eye health, sleep health, and physical activity addressed commonly recognized health consequences of excessive screen use; screen-time rules, mealtime screen use, and device placement addressed household routines and environmental control; and reinforcement sessions were included to support repeated exposure and caregiver implementation. Child-facing materials and home cues were included to make messages visible, concrete, and repeatedly encountered in daily routines.

## Table A2-1. Package Components

| Component | Target users | Intended function | Content and format | How presented during interviews |
|---|---|---|---|---|
| Caregiver education | Parents and intergenerational caregivers | Improve caregiver knowledge and practical supervision skills | Parent and grandparent lecture versions; offline lectures with online video replay; eight core topics | Short video clips from the caregiver lectures were shown through screen sharing |
| Child-facing materials | School-aged children and parent-child discussion | Translate key messages into age-adapted child-facing formats | Picture book and posters, age-adapted for 6-8, 9-12, and 13-14 years | Printed picture book was shown on camera; electronic posters were shown through screen sharing |
| Home reminder cues | Children and caregivers in daily household routines | Prompt screen-time rules at the point of daily decision-making | Table mat, door hanger, umbrella, sticky notes, and related reminder designs | Photographs of physical reminder cues were shown through screen sharing |

| | | | | |
|---|---|---|---|---|
| Home environment guidance | Caregivers and household decision-makers | Support self-assessment, graded feedback, and household environment modification | Online family screen environment self-assessment tool with six domains, 19 scored items, A-D grading, and rule-based tailored action suggestions | The online home-environment self-assessment tool was described verbally during the interview |

## Component 1. Caregiver Education

The caregiver education component was designed for adults responsible for children's daily routines and screen-use supervision. It included parent and grandparent versions to address both nuclear-family and intergenerational caregiving contexts. The component was intended to improve knowledge and practical supervision skills.

Eight core topics were included:

- Harms of excessive screen time
- Setting screen-time rules for school-aged children
- Screen time and eye health
- Screen time and sleep health
- Screen time and physical activity
- Mealtime screen use
- Placement and use of digital screen devices at home
- Reinforcement sessions to consolidate key messages

## Component 2. Child-Facing Materials

The child-facing component included a picture book and posters. The content was age-adapted for three groups: 6-8 years, 9-12 years, and 13-14 years. Materials for younger children emphasized simple rules, visual cues, and concrete daily situations; materials for older children included more explanation of consequences, self-management, autonomy, privacy protection, online strangers, and cyberbullying. The aim was to communicate screen-use guidance in language and formats that children could understand and discuss with caregivers. Because the caregivers interviewed in this formative evaluation had children aged 6 to 12 years, feedback on materials intended for children aged 13 to 14 years should be interpreted as indirect caregiver feedback and as a refinement issue for future pilot work.

## Table A2-2. Key Child-Facing Messages by Age Group

| Age group | Key child-facing messages |
|---|---|
| 6-8 years | Limit combined TV, mobile phone, and computer time to no more than 2 hours per day; rest after about 30 minutes; avoid screens before bed, while walking, during meals, and while talking with family; increase outdoor play; tell parents what was viewed online; follow family rules. |
| 9-12 years | Continue the 2-hour daily limit and breaks; avoid screens before bed, walking, meals, and conversations; protect privacy online; avoid contact with strangers online; explore constructive uses of digital devices beyond games; follow family rules. |
| 13-14 years | Limit screen time and take breaks; avoid screens before bed and avoid intense or frightening videos at night; avoid phone use while walking or cycling; maintain at least 1 hour of daily physical activity; avoid using phones while doing homework; protect privacy and safety online; avoid cyberbullying and seek help from trusted adults; use alternative activities when bored. |

## Component 3. Home Reminder Cues

The home reminder cues were intended to embed screen-time messages in daily routines and physical environments. Examples included:

- Umbrella: reminder not to look at a phone while crossing roads
- Table mat: reminder not to use screens during meals
- Door hanger: reminder not to use phones 1 hour before bedtime
- Sticky notes: reminder to look up and observe the sky

During the formative evaluation, caregivers commented on which reminder objects were likely to be noticed, kept, or used in daily routines.

## Component 4. Home Environment Guidance

The home-environment guidance component was operationalized as an online family screen-environment self-assessment tool for caregivers and household decision-makers. The tool assessed six domains of the family screen environment: physical environment, time rules, content supervision, joint screen use, caregiver modeling, and safety awareness. It combined self-assessment, graded feedback, and rule-based tailored action suggestions. It formed part of the caregiver-guidance and home-environment-support arms of the refined family action toolkit. The tool was designed to provide self-assessment and action suggestions only, not to claim effectiveness in reducing screen time.

## Table A2-3. Structure of the Home-Environment Self-Assessment Tool

| Element | Description | Purpose in the package |
|---|---|---|
| Entry questions | Child age group and caregiver or living arrangement. A hyperfocus or difficult-to-interrupt screening question can be used as a trigger item. | Enable age-adapted feedback and caregiver-structure-specific suggestions, including intergenerational or kinship caregiving contexts. |
| Scored items | Nineteen scored items, each scored from 1 to 4, with a maximum total of 76 points. Entry questions are not included in the total score unless the team decides to score the hyperfocus item. | Provide a structured family screen environment profile while avoiding claims of clinical diagnosis or intervention effect. |
| Assessment domains | Six domains: physical environment, time rules, content supervision, joint screen use, caregiver modeling, and safety awareness. | Translate broad screen-time guidance into concrete household dimensions that caregivers can recognize and act on. |
| Graded feedback | A-D family screen environment levels: A healthy type, B optimization needed, C attention needed, and D high-risk type. If the hyperfocus item is included in scoring, thresholds require recalibration. | Give caregivers an easy-to-understand profile of strengths and priority areas for improvement. |
| Tailored recommendations | Rule-based triggering of 3-5 prioritized recommendations from a recommendation library. Recommendations link to practical materials such as a charging station, table mat, reminder card, picture book, poster, refrigerator magnet, or family agreement template. | Convert assessment results into a short, feasible action list linked to home-embedded tools. |
| Action report | A family screen environment report containing the child age group, caregiver or living structure, total score, A-D level, six-domain radar profile, priority action list, weekly family agreement, reference resources, and disclaimer. | Close the loop between self-assessment, caregiver guidance, child-facing materials, and environmental support. |

## Table A2-4. Six Assessment Domains in the Self-Assessment Tool

| Domain | Number of items | Example focus areas |
|---|---|---|
| Physical environment | 6 | Bedroom devices, personal device ownership, total device density, main place of use, bedtime screen exposure, and screen-free areas. |
| Time rules | 3 | Weekday recreational screen time, weekend or holiday extension, and fixed device shutdown or storage time. |
| Content supervision | 3 | Caregiver awareness or joint selection of content, discussion of appropriate content, and short-video or social media use. |
| Joint screen use | 3 | Parent-child co-use, caregiver presence and interaction, and linking screen content to offline activities. |
| Caregiver modeling | 2 | Caregiver screen use in front of children and putting phones away during meals, play, or family time. |
| Safety awareness | 2 | Online privacy protection and caregiver awareness of online contacts, group chats, or game teammates. |

## Recommendation Logic for the Self-Assessment Tool

The recommendation library used rule-based triggers to match caregivers with a small number of high-priority suggestions. The revised specification included environmental restructuring, screen-time rule enforcement, content co-management, replacement activity planning, caregiver modeling, online safety conversations, device-return scripts, resistance-management scripts, parent-child agreement templates, intergenerational-caregiver consistency cards, age-adapted time guidance, and hyperfocus response strategies. The tool was designed to output 3-5 suggestions rather than an exhaustive list, so that caregivers would receive a manageable action plan. Recommendations used a consequence-chain plus supporting-material format where appropriate, linking the assessment result to a concrete household action and a corresponding reminder or discussion tool.

## Implementation Cautions Before Deployment

- All numerical thresholds, effect statements, examples, and cases should be checked by the research team against the adopted guidelines or cited sources before public deployment.
- Age-specific recreational screen-time thresholds should be finalized according to the guideline selected by the team.
- If the hyperfocus item is included in scoring, the total score and A-D grading thresholds should be recalibrated.
- Recommendations related to grandparents, kinship caregiving, or left-behind contexts should be reviewed to avoid stereotyping any family structure.
- Platform-specific functions such as youth mode or parental controls should be checked against current platform names and operating procedures at the time of deployment.
- The tool should remain positioned as a formative self-assessment and action-support tool; it should not claim to reduce screen time or replace medical, psychological, or educational advice.

## Planned Refinement Focus After Formative Evaluation

- Convert risk messages into concrete consequence chains linked to school, sleep, meals, road safety, and family interaction.
- Add more how-to guidance for rule-setting, parent-child negotiation, device-return strategies, and resistance management.
- Distinguish learning-related screen use from recreational screen use.
- Redesign home reminder cues as practical daily-use items that are less likely to be discarded.
- Strengthen age adaptation in child-facing materials, including readability, visuals, pinyin where appropriate, online safety, privacy, and autonomy for older children.
- Use the home-environment self-assessment tool to connect caregiver guidance, child-facing materials, home cues, and household environmental supports into a coherent family action toolkit.
