# Supplementary material for: Refining a Multicomponent Family-Based Health Education Package to Reduce Screen Time Among School-Aged Children: Qualitative Formative Evaluation With Parents and Caregivers
Source: JMIR Form Res. 2026 Oct 27;10:e106521. doi: 10.2196/106521 (PMC13712257; doi:10.2196/106521)
Supplement: Supplementary file 2 [file formative-v10-e106521-s002.pdf]

Multimedia Appendix 1

Semi-Structured Interview Guide for Evaluating the Health Education Materials

This English version was translated and lightly adapted from the original Chinese interview guide for reporting purposes. Only the package-evaluation section is presented here, as it was the section most directly relevant to the formative evaluation reported in this manuscript. In the interviews, participants reviewed selected examples rather than the complete package in full. The online home-environment self-assessment tool was described verbally rather than used interactively; feedback on this component was therefore interpreted as feedback on the tool's concept, intended function, and perceived usefulness.

Introduction to Participants

Next, we will show you selected examples from a preliminary family-based health education package about children's screen use and family supervision of screen time. The package includes caregiver education, child-facing materials, home-use reminder cues, and home-environment guidance. After each component is presented, we would like to hear your immediate impressions, what messages you remember most clearly, what parts are less memorable or less useful, and what revisions you suggest. There are no right or wrong answers. Please share your honest impressions. We are interested in how the materials are understood by caregivers and how they could be improved before pilot implementation. The components will be presented in the following order: caregiver education, child-facing materials, home-use reminder cues, and home-environment guidance. Short video clips, electronic posters, photographs of physical reminder materials, and the printed picture book will be shown during the interview. The online home-environment self-assessment tool will be described verbally rather than used interactively.

1. Information Uptake

After viewing or hearing about each component, what messages do you remember most clearly about children's screen use and family screen-time supervision?

Optional probes: For each component, did you notice or remember any information about the following topics? Which messages left a stronger impression, and which messages were less memorable?

- Excessive screen use and myopia
- Excessive screen use and obesity
- Excessive screen use and growth or development
- Excessive screen use and sleep
- Parent-child discussion and rule-setting
- Using electronic devices in shared or public spaces at home
- Not keeping electronic devices in the bedroom overnight
- Setting screen-free areas at home
- Limiting daily screen time to no more than 2 hours, preferably divided into 2 or 3 sessions, with each session no more than 30 minutes
- Keeping an appropriate viewing distance from televisions or computers
- Avoiding mobile phones or tablets 1 hour before bedtime
- Not looking at a phone while walking
- Hyperfocus or becoming absorbed in screens
- Replacing screen use with parent-child activities, outdoor activities, or household activities

2. Comprehensibility

1. Were the materials easy to understand?
2. Were there any words, sentences, images, or explanations that you did not understand?
3. Was there any part of the materials whose meaning was unclear?
4. How could that part be explained more clearly?

3. Conciseness and Information Load

5. Did you feel that any part of the materials was unnecessary or repetitive?

6. Did the materials contain too much information, too little information, or an appropriate amount of information?
7. Was the pace of the materials appropriate?
8. What information should be shortened, emphasized, repeated, or added?

4. Perceived Guidance and Usefulness

9. Do you think these materials could influence you in any way? For example, your understanding of the harms of excessive screen time, your views on screen supervision, or what you may pay attention to in the future.
10. After viewing the materials, do you think you might make any changes in how you supervise your child's screen use?
    - If yes, what changes?
    - If no, why not?
11. Which parts of the materials are most useful for helping parents take action at home?
12. Which parts are not practical or not specific enough?
13. What additional guidance would help parents act more effectively? For example, rule-setting methods, parent-child negotiation, managing resistance, controlling duration, distinguishing learning-related and recreational screen use, or providing alternative activities.

5. Attractiveness and Engagement

14. Did you like the materials? Why or why not?
15. Were the materials attractive or engaging?
16. Which parts were more memorable?
17. Which parts were less impressive or less engaging?
18. How could the materials be made more attractive to parents or children?

6. Overall Evaluation

19. Overall, what do you think of these health education materials?
20. What do you think of the format, visual design, wording, and presentation style?
21. What are the main strengths of the materials?
22. What are the main weaknesses?
23. What would you most want to revise before these materials are used more widely?

7. Appropriateness of Material Type and Delivery Format

24. Do you think these materials are suitable for parents or caregivers like you?
25. Do you think these materials are suitable for children in your child's age group?
26. Which format do you think is most suitable? For example, video clips, lectures, picture books, posters, brochures, school-based activities, mobile applications, short videos, parent meetings, or home-use reminder materials.
27. Where do you think these materials should be used or displayed? For example, at home, in schools, in communities, on public screens, on social media, or during parent meetings.
28. Do you have any suggestions for improving the material type, delivery channel, or target audience?

Closing Question

Is there anything else you would like to share about these health education materials?
