# Supplementary material for: Refining a Multicomponent Family-Based Health Education Package to Reduce Screen Time Among School-Aged Children: Qualitative Formative Evaluation With Parents and Caregivers
Source: JMIR Form Res. 2026 Oct 27;10:e106521. doi: 10.2196/106521 (PMC13712257; doi:10.2196/106521)
Supplement: Supplementary file 3 [file formative-v10-e106521-s003.pdf]

Multimedia Appendix 3. Coding Framework and Theme Development

Refining a Multicomponent Family-Based Health Education Package to Reduce Screen Time Among School-Aged Children: Qualitative Formative Evaluation

1. Analytic approach

Transcripts from the package-evaluation section were analyzed using directed content analysis and reflexive thematic analysis. Directed content analysis provided the initial deductive coding structure, which was derived from the Theoretical Framework of Acceptability (TFA) and health communication material-evaluation dimensions. Reflexive thematic analysis was then used to identify patterns that were not fully captured by the initial framework. Coding focused on material and package-evaluation content; general family screen-time supervision experience was retained only as background context unless it was directly linked to participants' evaluation of the package.

2. Coding framework (codebook)

Each meaning unit was coded with one or more framework codes, tagged for the component it referred to, and flagged for scope. The deductive framework included TFA constructs and health communication material-evaluation dimensions. Inductive codes were added when participants raised issues not sufficiently specified in the initial framework, such as hyperfocus as a recognizable family scenario, difficulty distinguishing learning-related and recreational screen use, conflict around returning devices, the need for replacement activities, and preferences for routine-embedded objects such as table mats, stationery, and notebooks. Definitions used by both coders are shown below.

2.1 Acceptability codes (Theoretical Framework of Acceptability)

Code	Construct	Definition (when applied)
TFA-AA	Affective attitude	How the caregiver feels about a component (liking, emotional response, resonance).
TFA-BU	Burden	Perceived effort or difficulty in understanding or using the material.
TFA-ET	Ethicality	Fit between the material and the caregiver's values (eg, privacy, autonomy).
TFA-CO	Intervention coherence	Whether the caregiver understands how a component works and its purpose.
TFA-OC	Opportunity cost	What must be given up (time, money, effort) to adopt the material or advice.
TFA-PE	Perceived effectiveness	Belief that the material will help achieve the intended goal.
TFA-SE	Self-efficacy	Confidence in being able to enact what the material suggests.

2.2 Health communication material-evaluation dimensions

Code	Dimension	Definition (when applied)
MAT-UP	Information uptake / recall	What messages were taken in, retained, or missed.
MAT-CM	Comprehensibility	Clarity and ease of understanding.
MAT-CN	Conciseness & pacing	Length or pacing (too long or short; presentation speed).
MAT-GU	Perceived guidance / actionability	Whether the material tells caregivers how to act.
MAT-AT	Attractiveness / appeal	Visual design, color, animation quality, and emotional impact.
MAT-OI	Overall impression	Global evaluation of a component or the package.
MAT-AP	Appropriateness / fit	Age, caregiver, format, or context fit; ambiguous wording.
MAT-SG	Suggestions for improvement	Explicit requests for changes.

2.3 Component tags and scope

- Component tags: C-LEC (caregiver lecture / video), C-CHILD (picture book and posters), C-CUE (home reminder cues), C-ENV (home-environment self-assessment tool), C-GEN (whole package or unspecified).
- Scope: A = material/package evaluation (analyzed); B = general supervision experience (background only).

3. Theme development

Framework codes and inductively developed patterns were grouped into four themes according to the main refinement problem they identified. Theme 1 was primarily informed by deductive codes on acceptability, comprehension, and self-reported immediate recall. Themes 2 and 3 combined deductive material-evaluation codes with inductive codes on concrete scenarios, consequence chains, and action guidance. Theme 4 was mainly developed from inductive codes on age adaptation, daily-use objects, and fit with household routines, while also drawing on deductive codes related to appropriateness, burden, and perceived usefulness. The table shows each theme, its definition, and the codes that most often contributed to it.

Theme	Definition	Primary contributing codes
Theme 1. Generally acceptable, but immediate recall varied	Materials were understandable and acceptable, but some messages were not consistently recalled after exposure (self-reported during interviews).	MAT-CM, MAT-UP (with TFA-CO)
Theme 2. Concrete, scenario-based messages were described as more meaningful than abstract risks	Messages tied to familiar family or school scenarios were described as more meaningful; caregivers asked for concrete consequence chains and real-life cases.	TFA-AA, MAT-AT, MAT-SG
Theme 3. Caregivers wanted action guidance rather than additional risk information	Caregivers reported already understanding the harms but wanted practical guidance on how to act (rules, duration control, negotiation, managing resistance, alternatives).	MAT-GU, TFA-SE (with MAT-SG)
Theme 4. Practical, age-adapted, home-embedded tools were preferred	Caregivers preferred high-frequency, practical, age-adapted materials embedded in daily routines, and environmental supports.	MAT-AP, MAT-SG, TFA-PE
(T0) Background / not assigned	Content reflecting general supervision experience or unrelated to package evaluation.	Not applicable

4. Coding procedure and reconciliation

- Two researchers independently read the transcripts and applied the initial coding framework.
- ChatGPT, using GPT-5.5 Thinking through the OpenAI web interface, was used in June and July 2026 as a supplementary organizational aid. The tasks assigned to the tool were limited to organizing de-identified qualitative excerpts by provisional topic, and supporting English-language editing.
- Selected de-identified meaning units from the package-evaluation section were submitted to the AI tool. These consisted of short excerpt-level units rather than full transcripts and were used only for provisional grouping and language organization. Full transcripts, audio recordings, participant names, contact information, locations, institutions, and the raw reconciliation spreadsheet were not submitted.
- AI-generated suggestions were treated as provisional organizational outputs, not as evidence or independent analytic confirmation. Suggestions were checked against the original transcripts by two researchers and were corrected, discarded, or incorporated only when supported by the data.
- The two researchers compared their coding, discussed discrepancies, refined the codebook, finalized themes, and selected quotations directly from verified transcripts.

Pre-reconciliation coding comparison was summarized at three levels. The double-coded comparison set comprised 40 meaning units from the package-evaluation corpus. These figures are reported to make the reconciliation process transparent; they are not presented as evidence of statistical reliability or validity. Participant-level coverage counts below are based on the full coded corpus.

Level of comparison	Pre-reconciliation result	Interpretation
Scope (A/B)	40/40 units agreed (100%)	Both coders agreed on whether each unit belonged to material/package evaluation or background context.
Component tagging	36/40 units agreed (90%)	Component tagging agreed for the large majority of units; differences were resolved during codebook clarification.
Theme assignment	24/40 units agreed (60%); Cohen's κ = 0.53	All theme-level disagreements concerned whether a unit should be assigned to a substantive theme versus background/not assigned. No disagreement involved two different substantive themes.
Framework-code granularity	Qualitative comparison only	Framework-code differences reflected whether coders applied a single core code or all applicable codes, not conflicting interpretations of the same excerpt.

All discrepancies were reviewed and resolved through discussion. Two codebook clarifications were adopted: (1) in-scope evaluative units are assigned to the relevant theme, and only non-evaluative background is left unassigned; and (2) all applicable codes are applied to each unit. One unit reflecting an interpretive difference (a comment about a newly encountered concept, hyperfocus) was coded as affective attitude by consensus.

The two coders never diverged on which substantive theme or which dimension was relevant; disagreements concerned only whether a unit was theme-relevant versus background, and coding granularity. These were resolved through the two codebook clarifications, after which coding converged. Here, "converged" refers to consensus resolution after discussion and does not refer to a simulated or post-reconciliation reliability statistic. No projected or simulated kappa values were used or reported.

The raw reconciliation spreadsheet is not included as a public appendix because it contains original-language excerpts, timestamps, and participant-level identifiers. This appendix presents only a de-identified summary of the coding framework, reconciliation process, and evidence coverage.

5. Evidence coverage

To show the spread of supporting evidence across participants, participant-level coded evidence is summarized below. Filled cells indicate that a participant contributed at least one coded excerpt to a theme; empty cells indicate that no relevant coded excerpt was identified. These cells summarize evidence coverage and should not be interpreted as prevalence estimates, subgroup comparisons, or evidence of generalizability.

Theme	Participants with coded evidence
Theme 1. Generally acceptable, but immediate recall varied	9/10
Theme 2. Concrete, scenario-based messages were described as more meaningful than abstract risks	7/10
Theme 3. Caregivers wanted action guidance rather than additional risk information	7/10
Theme 4. Practical, age-adapted, home-embedded tools were preferred	8/10

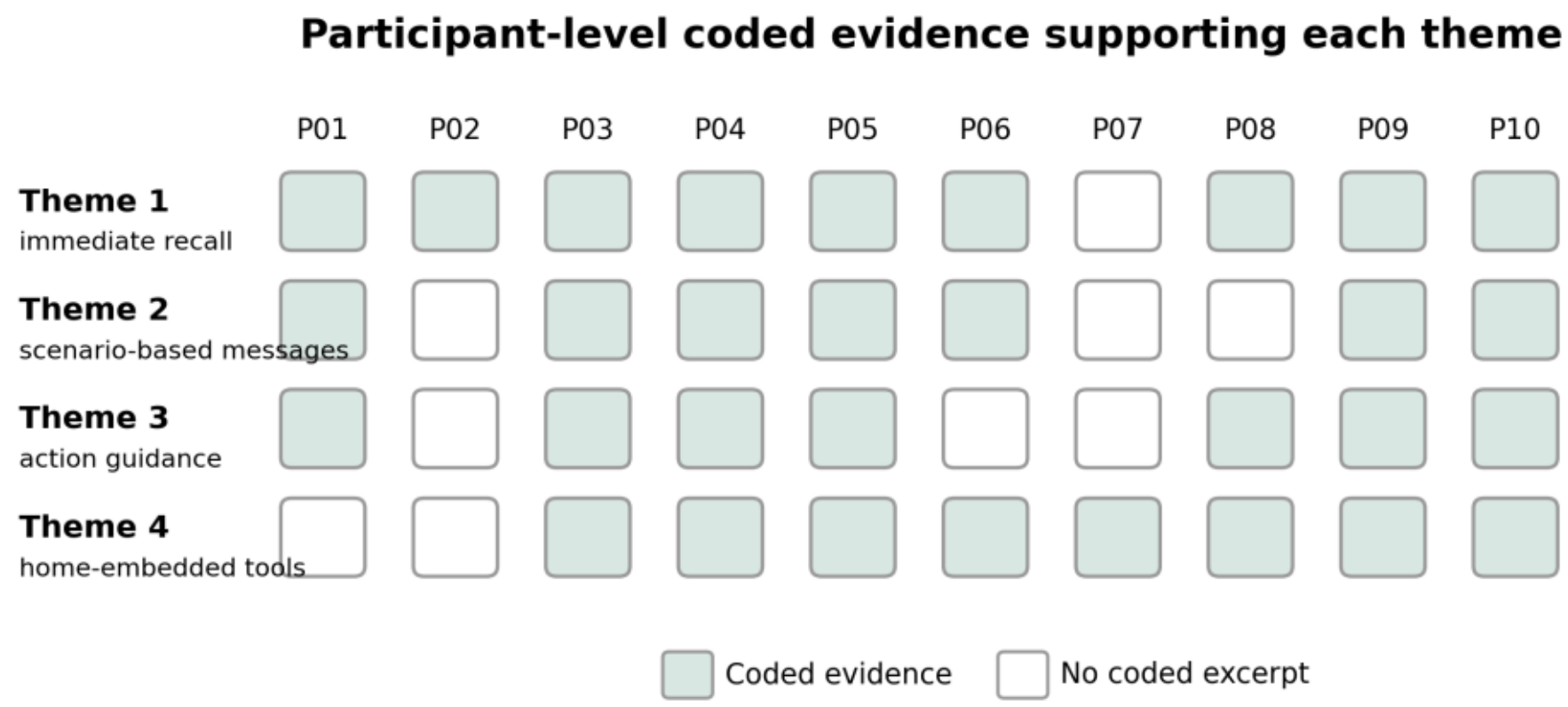

Supplementary Figure 1. Participant-Level Coded Evidence

Filled cells indicate that the participant contributed at least one coded excerpt supporting the theme; empty cells indicate that no relevant coded excerpt was identified. The matrix summarizes evidence coverage and should not be interpreted as quantitative prevalence estimates.

6. Interpretive cautions

- The coded counts indicate coverage of evidence across participants rather than statistical prevalence, subgroup difference, or generalizability.
- Positive responses, stated willingness, and perceived usefulness were interpreted as formative feedback, not as evidence of intervention effectiveness.
- The themes describe caregivers' perceptions of selected examples from a researcher-presented preliminary package shown during online interviews. They may differ from actual use of the complete package in home, school, or community implementation.
- Feedback on the online home-environment self-assessment tool should be interpreted as feedback on the tool's concept, intended function, and perceived usefulness because the tool was described verbally rather than used interactively during interviews.
- Feedback on materials intended for children aged 13 to 14 years should be interpreted cautiously because the interviewed caregivers had children aged 6 to 12 years.
