# Supplementary material for: Refining a Multicomponent Family-Based Health Education Package to Reduce Screen Time Among School-Aged Children: Qualitative Formative Evaluation With Parents and Caregivers
Source: JMIR Form Res. 2026 Oct 27;10:e106521. doi: 10.2196/106521 (PMC13712257; doi:10.2196/106521)
Supplement: Supplementary file 4 [file formative-v10-e106521-s004.pdf]

Multimedia Appendix 4

Additional Quotations

Quotations were selected from verified Chinese transcripts, anonymized, translated into English by a bilingual member of the research team, and checked against the original Chinese transcripts by a second bilingual researcher. Quotations were lightly edited for readability only when necessary, for example by removing fillers, repeated words, or incomplete sentence fragments, without changing participants' meaning or voice. Omissions are indicated with ellipses, and clarifying words are inserted in brackets where needed to preserve readability or protect anonymity. This appendix provides additional supporting quotations beyond those highlighted in the main manuscript table.

Theme	Participant	Additional quotation	Analytic relevance
Theme 1	P01	"…easy to understand. Even if I took it out and showed it to [the child], [the child] could understand it."	Acceptability and suitability for parent-child viewing
Theme 1	P03	"After watching it, I felt a little confused."	Immediate recall was not always strong despite comprehensibility
Theme 1	P09	"I suddenly forgot."	Some participants had difficulty recalling specific harm messages
Theme 1	P10	"I forgot…I cannot recall it, but I roughly know the general idea."	Participants sometimes retained a broad impression without details
Theme 3	P07	"Previously I did not know how long I should control [the child's] screen time each day…[the poster] gave a clear standard."	Need for concrete duration standards and actionable guidance
Theme 2	P10	"Hyperfocus…that is very important."	Hyperfocus was identified as a salient concept
Theme 2	P03	"During the summer holiday, [the child] did have this once…watching very intensely…I did not know how to solve it."	Hyperfocus linked to familiar family experience and management uncertainty
Theme 2	P01	"The harms are definitely myopia, and sleep is indeed affected…also concentration."	Visual, sleep, and concentration harms were more readily named
Theme 2	P06	"The impact was not strong…I need something more impactful, whether in the images or the content."	Some participants wanted stronger, more vivid risk communication
Theme 3	P05	"At the end of the video, there could be more concrete measures, such as activities that are common, interesting to children, and do not cost much."	Replacement activities needed to be specific, attractive, and low-cost
Theme 3	P01	"What method could help [the child] return the electronic device voluntarily at the agreed time?"	Caregivers wanted device-return strategies
Theme 3	P04	"[Replacement activities] could be richer…I only heard about outdoor activities and trips, not much else."	Requests for broader alternatives to screen use
Theme 3	P09	"Everyone understands it, but it is very hard to do."	Implementation difficulty despite awareness
Theme 4	P08	"I think these things are quite useful…when parents forget, seeing them may remind them to manage [the child]."	Reminder cues may support caregiver action
Theme 4	P07	"If it is really a picture book, it may be more suitable for preschool children."	Age-fit concerns for child-facing formats
Theme 4	P10	"[The picture book] is attractive, but it needs pinyin…children can only look at the pictures; they may not know the words."	Readability and literacy adaptation
Theme 2	P03	"The small text under the poster was very small; I could not see it clearly…It could use cartoons or pictures and explain why."	Need for more vivid, visually clear explanations and concrete reasoning
Theme 4	P07	"External channels are only publicity reminders…more important is that parents and [the child] reach an agreement."	Reminder materials should support family agreement rather than replace family negotiation

Notes on Quotation Handling

- Ellipses indicate omitted words, fillers, repeated speech, or transitions that did not change the meaning.
- Clarifying words are inserted in brackets where needed to preserve readability or protect anonymity.
- References to children, locations, institutions, specific platforms, learning devices, games, and picture-book titles were anonymized where needed.
- The quotations support participants' perceptions of package acceptability, usefulness, and refinement needs. They do not indicate intervention effectiveness.
