# Supplementary material for: Refining a Multicomponent Family-Based Health Education Package to Reduce Screen Time Among School-Aged Children: Qualitative Formative Evaluation With Parents and Caregivers
Source: JMIR Form Res. 2026 Oct 27;10:e106521. doi: 10.2196/106521 (PMC13712257; doi:10.2196/106521)
Supplement: Supplementary file 5 [file formative-v10-e106521-s005.pdf]

Multimedia Appendix 5

COREQ Checklist

COREQ indicates Consolidated Criteria for Reporting Qualitative Research. This checklist is completed for the qualitative formative evaluation manuscript. Page and line numbers will be added at the proof stage.

COREQ item	Status	Where addressed	Notes
**Domain 1: Research team and reflexivity**			
1. Interviewer/facilitator	Reported	Data Collection	All interviews were conducted by a single graduate-student interviewer.
2. Credentials	Reported	Data Collection	The interviewer was a graduate student in public health who had received training in qualitative interviewing.
3. Occupation	Reported	Data Collection	Graduate student on the project team.
4. Gender	Reported	Data Collection	The interviewer was a woman.
5. Experience and training	Reported	Data Collection	Interviewer had received qualitative interviewing training.
6. Relationship established	Reported	Data Collection	Participants had no prior relationship with the interviewer.
7. Participant knowledge of interviewer	Reported	Data Collection	Before providing consent, participants were told the study purpose and procedures and that the interviewer was a graduate student conducting research on the screen-time education materials.
8. Interviewer characteristics	Partially reported	Data Collection; Competing Interests	The interviewer was not a member of the material-development team and had no prior relationship with participants; coders, including the interviewer, documented prior assumptions (see Trustworthiness).
**Domain 2: Study design**			
9. Methodological orientation and theory	Reported	Study Design; Data Analysis	Directed content analysis and reflexive thematic analysis. The initial deductive framework was informed by the Theoretical Framework of Acceptability and health communication material-evaluation dimensions; inductive coding was used to identify patterns not fully captured by the initial framework.
10. Sampling	Reported	Participants and Recruitment	Purposive selection of 10 caregivers from 167 interested caregivers using a sampling matrix. The matrix considered region, caregiver role, caregiver age, number of children, household size, family structure, and whether grandparents or other relatives were involved in caregiving. Sampling sought variation in caregiving contexts, not subgroup comparison.
11. Method of approach	Reported	Participants and Recruitment	Study announcement posted on Wenjuanxing online survey platform.
12. Sample size	Reported	Participants and Recruitment; Results	Ten parents or primary caregivers. The target sample size of approximately 10 interviews was determined in advance for a focused, package-specific formative evaluation and was justified using the information power approach.
13. Non-participation	Reported	Participants and Recruitment	167 caregivers expressed interest; 10 were purposively selected using the sampling matrix. The remaining interested caregivers were not invited because the study used a small, focused formative-evaluation sample rather than open enrollment. Reasons for refusal after invitation were not reported.
14. Setting of data collection	Reported	Data Collection	Online video calls on Tencent Meeting.
15. Presence of non-participants	Reported	Data Collection	Interviews were conducted one-to-one online; no non-participants were present.
16. Description of sample	Reported	Participants and Recruitment; Table 1	Caregiver type, child school stage, family context, and supervision context.
17. Interview guide	Reported	Data Collection; Multimedia Appendix 1	Package-evaluation section of the semi-structured interview guide provided in Multimedia Appendix 1. The guide covered information uptake, comprehensibility, conciseness, perceived guidance, attractiveness, overall impression, appropriateness, and suggestions for improvement.
18. Repeat interviews	Reported	Data Collection	No repeat interviews were conducted.
19. Audio/visual recording	Reported	Data Collection; Declarations	Interviews were audio-recorded with oral informed consent.
20. Field notes	Reported	Data Collection	Interviewer took field notes during and immediately after interviews.

COREQ item	Status	Where addressed	Notes
21. Duration	Reported	Data Collection	Each interview lasted approximately 60 minutes; the package-evaluation section occupied most of the interview time and usually lasted approximately 45 to 60 minutes.
22. Data saturation/information power	Reported	Participants and Recruitment	Sample judged sufficient based on information power for a narrow, package-specific formative aim. After 10 interviews, feedback repeatedly converged around actionability, concrete examples, age adaptation, and home-embedded supports.
23. Transcripts returned	Reported (not performed)	Data Analysis	Transcripts were not returned to participants for comment or correction.
**Domain 3: Analysis and findings**			
24. Number of data coders	Reported	Data Analysis; Trustworthiness	Two researchers independently read the transcripts and applied the initial coding framework before reviewing any AI-assisted output. Coding was compared and reconciled through discussion.
25. Description of coding tree	Reported	Data Analysis; Multimedia Appendix 3	Complete coding framework, deductive and inductive code development, theme development, reconciliation information, and participant-level evidence coverage are provided in Multimedia Appendix 3.
26. Derivation of themes	Reported	Data Analysis; Results; Multimedia Appendix 3	Themes were developed by grouping deductive and inductive codes according to the main refinement problem they identified: acceptability and recall, concreteness of messages, actionability of caregiver guidance, and practical fit of tools in family routines.
27. Software	Reported	Data Analysis	Microsoft Word and Excel were used to manage coded excerpts, code definitions, analytic memos, theme matrices, and package-refinement recommendations. ChatGPT, using GPT-5.5 Thinking through the OpenAI web interface, was used in June and July 2026 only as a supplementary organizational and language-editing aid.
28. Participant checking	Reported (not performed)	Data Analysis	Member checking was not conducted.
29. Quotations presented	Reported	Results; Table 2; Multimedia Appendix 4	Representative quotations are provided in Table 2, and additional quotations are provided in Multimedia Appendix 4. Quotations were selected from verified Chinese transcripts, anonymized, translated into English by a bilingual researcher, and checked by a second bilingual researcher.
30. Data and findings consistent	Reported	Results; Tables 2-3; Multimedia Appendices 3-4	Themes are supported by representative quotations, additional quotations, participant-level evidence coverage, and component-level refinement implications. Participant counts indicate evidence coverage rather than prevalence or generalizability.
31. Clarity of major themes	Reported	Results	Four final themes are named and described.
32. Clarity of minor themes	Reported	Results; Multimedia Appendix 3	Analysis focused on four major formative themes rather than formal subtheme reporting. Component-level refinement implications are summarized in Table 3, and coding details are provided in Multimedia Appendix 3.
